# PIEZO1 Channel Is a Potential Regulator of Synovial Sarcoma Cell-Viability

**DOI:** 10.3390/ijms19051452

**Published:** 2018-05-14

**Authors:** Takahisa Suzuki, Yukiko Muraki, Noriyuki Hatano, Hiroka Suzuki, Katsuhiko Muraki

**Affiliations:** Laboratory of Cellular Pharmacology, School of Pharmacy, Aichi-Gakuin University, 1-100 Kusumoto, Chikusa, Nagoya 464-8650, Japan; c172903@ed.nagoya-cu.ac.jp (T.S.); ymuraki@helen.ocn.ne.jp (Y.M.); nhatano@dpc.agu.ac.jp (N.H.); hsuzuki@dpc.agu.ac.jp (H.S.)

**Keywords:** PIEZO1, Yoda1, synovial sarcoma, mechanical stress, cell-viability

## Abstract

Detection of mechanical stress is essential for diverse biological functions including touch, audition, and maintenance of vascular myogenic tone. PIEZO1, a mechano-sensing cation channel, is widely expressed in neuronal and non-neuronal cells and is expected to be involved in important biological functions. Here, we examined the possibility that PIEZO1 is involved in the regulation of synovial sarcoma cell-viability. Application of a PIEZO1 agonist Yoda1 effectively induced Ca^2+^ response and cation channel currents in *PIEZO1*-expressing HEK (HEK-Piezo1) cells and synovial sarcoma SW982 (SW982) cells. Mechanical stress, as well as Yoda1, induced the activity of an identical channel of conductance with 21.6 pS in HEK-Piezo1 cells. In contrast, Yoda1 up to 10 μM had no effects on membrane currents in HEK cells without transfecting *PIEZO1*. A knockdown of *PIEZO1* with siRNA in SW982 cells abolished Yoda1-induced Ca^2+^ response and significantly reduced cell cell-viability. Because *PIEZO1* is highly expressed in SW982 cells and its knockdown affects cell-viability, this gene is a potential target against synovial sarcoma.

## 1. Introduction

Detection of mechanical stress is essential for diverse biological functions including touch, audition, and maintenance of vascular myogenic tone. In the latter, shear stimuli into vascular endothelial cells can activate various endothelial ion channels and subsequently affect vascular functions. Therefore, mechano-sensors in the endothelium are important regulators of blood pressure, arteriosclerosis, and vascular remodeling. We and others have recently identified that vascular endothelial cells express *PIEZO1*, a mechano-sensing cation channel that regulates vascular development in neonatal mice [1,2]. Because *PIEZO1* is widely expressed in neuronal and non-neuronal cells, we anticipate it to be involved in other important biological functions [3].

The PIEZO family of cation selective channels includes two isoforms, *PIEZO1* and *PIEZO2* [4]. Both PIEZO1 and PIEZO2 are directly activated by mechanical stress [5]. PIEZO1 is also activated by shear stimuli on the cell membrane [1] and a chemical agonistic compound, 2-[5-[[(2,6-Dichlorophenyl)methyl]thio]-1,3,4-thiadiazol-2-yl]-pyrazine (Yoda1) [6]. PIEZO1 is a sensor in the integrated response to bladder distention in urothelium [7] and contributes to mechanical stress-induced osteoclastogenesis in human periodontal ligament cells [8]. Moreover, PIEZO1 is involved in red blood cell function, because mutations in this gene cause dehydrated hereditary xerocytosis [9,10,11,12]. In turn, *PIEZO2* is predominantly expressed in sensory tissues. In particular, it is a mechano-sensor in Merkel cells and plays a key role in mediating the moderate touch sensation on the skin [13,14,15]. Although extensive studies have been performed after PIEZO1 and PIEZO2 were found to be mechano-sensors, functional analyses of these proteins are still limited.

In the present study, we examined the possibility that PIEZO1 is involved in the regulation of synovial sarcoma cell-viability. Synovial sarcoma is a malignant neoplasm that can arise at almost any anatomic site and accounts for 10–20% of soft-tissue sarcomas in adolescents and young adults [16]. Although cytotoxic chemotherapy against synovial sarcoma with drugs such as ifosfamide and anthracyclines is potentially promising [17,18], the emergence of drug resistance during the treatment is common. Therefore, novel therapeutic strategies and new cytotoxic drugs are required. Because PIEZO1 is also known as Fam38A, an integrin-interacting protein [19], we hypothesized that its dysfunction may affect cancer cell survival. Here, by employing PIEZO1 agonist Yoda1 and siRNA technology, we demonstrate that *PIEZO1* is highly expressed in human synovial sarcoma SW982 cells and its knockdown affects the cell-viability.

## 2. Results

### 2.1. Effects of Yoda1 on HEK-Piezo1 and SW982 Cells

**Figure 1:**
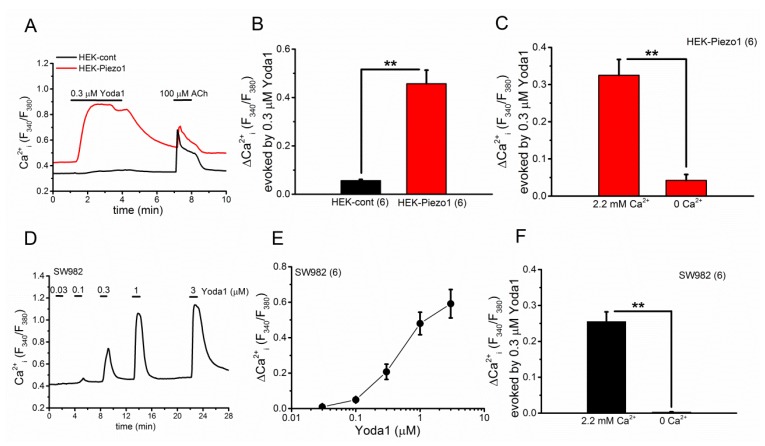
Effects of Yoda1 on HEK-Piezo1 (**A**–**C**) and SW982 cells (**D**–**F**). (**A**) A representative Ca^2+^ response of HEK-Piezo1 and HEK-cont cells to 0.3 μM Yoda1 and 100 μM Ach; (**B**) A summary of the peak change in Ca^2+^ response of HEK-Piezo1 and HEK-cont cells to Yoda1; (**C**) A summary of the peak evoked Ca^2+^ response of HEK-Piezo1 cells in the presence and absence of extracellular Ca^2+^ to Yoda1 (0.3 μM); (**D**) A representative Ca^2+^ response of SW982 cells to a range of Yoda1 concentrations between 0.03 and 3 μM; (**E**) The peak change of Ca^2+^ response of SW982 cells to a range of Yoda1 concentrations; (**F**) A summary of the peak evoked Ca^2+^ response of SW982 cells in the presence and absence of extracellular Ca^2+^ to Yoda1 (0.3 μM). Pooled data are averaged and expressed as mean ± SEM. Statistical significance was established using Student’s *t*-test. ** *p* < 0.01 compared with each corresponding control group. The numbers in parentheses indicate the number of independent experiments.

To re-examine the effectiveness of Yoda1 on the PIEZO1 channel, we applied Yoda1 to HEK cells transiently expressing human *PIEZO1* (HEK-Piezo1). As shown in Figure 1A,B, the application of 0.3 μM Yoda1 evoked Ca^2+^ response in HEK-Piezo1 cells, while it had little effect on native HEK cells (HEK-cont), where only *PIEZO1* was expressed at a low level. In addition, the Ca^2+^ response of HEK-Piezo1 cells to Yoda1 was significantly attenuated in SBS without Ca^2+^ (Figure 1C), confirming that Yoda1 is an effective activator of human PIEZO1 as previously reported [6,20]. We next examined the effects of Yoda1 on synovial sarcoma SW982 cells. As shown in Figure 1D,E, the application of Yoda1 at a range of concentrations from 0.03 to 3 μM evoked Ca^2+^ response in SW982 cells in a concentration-dependent manner. Moreover, the removal of Ca^2+^ from SBS abolished Ca^2+^ response to 0.3 μM Yoda1 (Figure 1F), suggesting that SW982 cells highly express the PIEZO1 channel.

### 2.2. PIEZO1 Channel Activation by Yoda1 and Mechanical Stress

**Figure 2:**
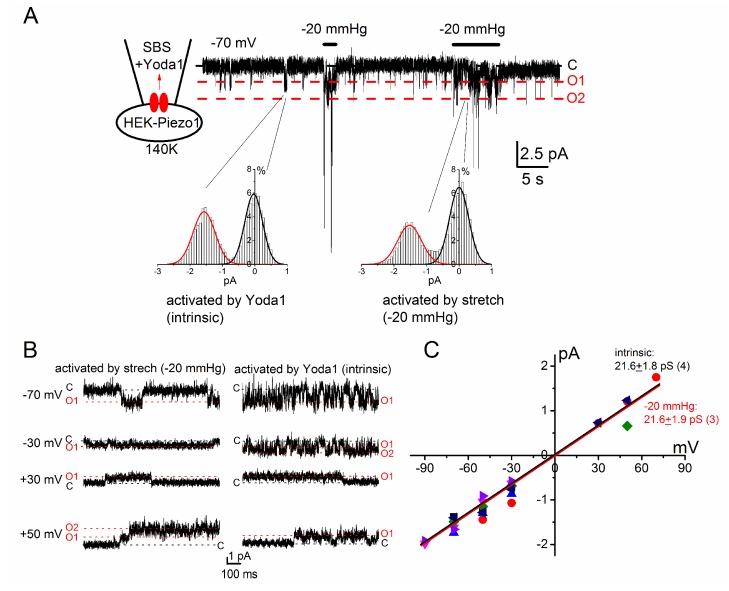
Comparison between Yoda1- and stretch-induced channel activities in HEK-Piezo1 cells. Channel activity was recorded in the cell-attached patch configuration (see the schematic inset and Methods and Materials in details). (**A**) The representative channel activity at a holding potential of −70 mV, evoked intrinsically and induced by a negative pressure of 20 mmHg to the patch membrane. Amplitude histograms for a 1-s period during activation of the intrinsic opening channel and stretch-induced channel, respectively, are shown in insets; (**B**,**C**) The channel activity induced by Yoda1 (intrinsic) and stretch (−20 mmHg) compared at different holding potentials. The ‘C,’ ‘O1’, and ‘O2’ labels denote activity levels corresponding to both channels closing, the first channel opening, and the second channel opening, respectively. These data were obtained from two different cells; (**C**) The unit amplitude of each channel summarized against potentials. The numbers in parentheses indicate the number of independent experiments.

**Figure 3:**
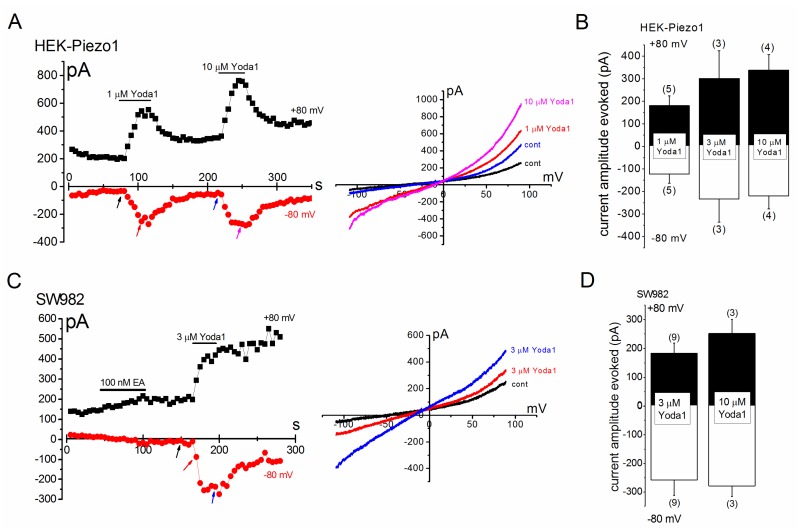
Yoda1-induced cation currents in HEK-Piezo1 and SW982 cells. (**A**,**B**) HEK-Piezo1 cells were voltage-clamped in whole-cell configuration mode and treated with 1 μM or 10 μM Yoda1. (**A**) Left panel: Ramp waveform pulses from −110 to +90 mV for 400 ms were applied every 5 s and the peak amplitude of cation currents at −80 and +80 mV was plotted against time. Arrows denote the time at which each I-V was detected. Right panel: A typical I-V exhibited before and after application of 1 μM and 10 μM Yoda1; (**B**) A summary of the peak amplitudes of cation currents at −80 and +80 mV in the presence of Yoda1; (**C**,**D**) SW982 cells were treated with Yoda1 under similar experimental conditions to HEK-Piezo1 cells. SW982 cells were exposed to EA at 100 nM as previously reported. Pooled data are averaged and expressed as mean ± SEM. The numbers in parentheses indicate the number of independent experiments.

Because both Yoda1 and mechanical stimuli activate the PIEZO1 channel, we examined electrophysiological profiles of Yoda1-induced cation currents in HEK-Piezo1 and SW982 cells (Figure 2 and Figure 3). In a cell-attached patch clamp configuration, where the pipette solution contained SBS with 3 μM Yoda1, intrinsic channel activity was found at −70 mV (−1.55 ± 0.08 pA, *n* = 4, also see the histogram on the left in the inset). The application of an additional negative pressure of 20 mm Hg to the patch membrane induced a higher channel activity, whose unit current (−1.52 ± 0.08 pA, *n* = 3, see also the histogram on the right in the inset) was similar to the intrinsic one. Figure 2B,C show Yoda1- and mechanical stress-sensitive channel activity against a range of holding potentials and a summary of each activity, respectively, suggesting that Yoda1 and mechanical stress both activate the PIEZO1 channel. In Figure 3, Yoda1 was applied to HEK-Piezo1 (Figure 3A,B) and SW982 cells (Figure 3C,D) under whole-cell conditions. Yoda1 effectively activated cation channel currents in HEK-Piezo1 and SW982 cells. In both cell types, the activation was largely reversible at a negative potential after the wash-out of Yoda1, whereas the activation was partially irreversible at a positive potential. In contrast, HEK-cont cells were insensitive to 10 μM Yoda1 (Supplementary Figure S1B). Moreover, HEK cells transfected with a canonical human Transient Receptor Potential Channel 4 (HEK-hTRPC4) were also resistant to 3 μM Yoda1, while these cells responded to 100 nM EA, an TRPC4 agonist (Supplementary Figure S1A,B, [21]). These data strongly suggest that SW982 cells express functional PIEZO1 channels which are effectively activated by Yoda1.

### 2.3. Knocking down of PIEZO1 Channel in SW982 Cells

**Figure 4:**
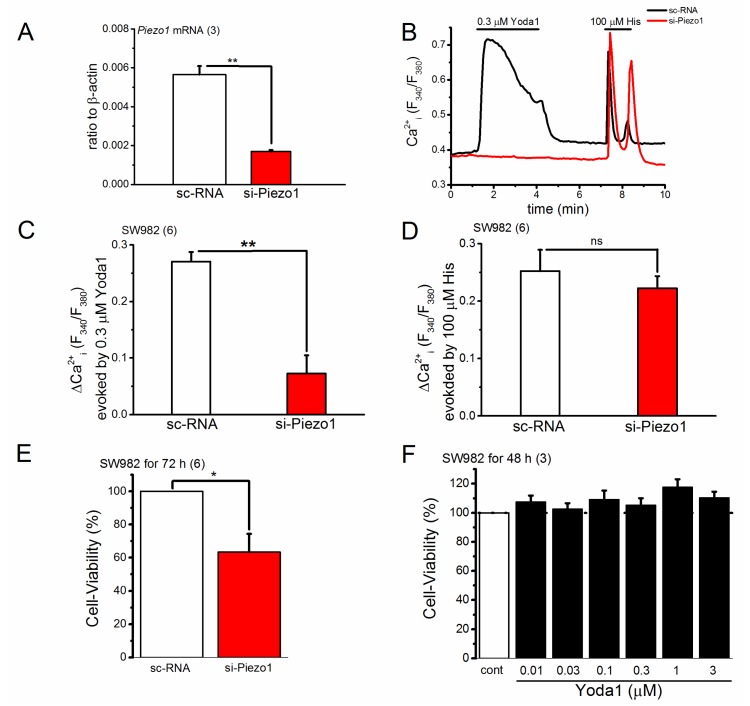
*PIEZO1* knockdown in SW982 cells and its effect on the cell-viability. (**A**–**D**) The expression of *PIEZO1* in SW982 cells was reduced by si-Piezo1 treatment for 72 h. (**A**) The mRNA expression level of *PIEZO1* in SW982 cells transfected with si-Piezo1 compared with that in cells transfected with stealth control siRNA (sc-RNA); (**B**) A representative Ca^2+^ response to Yoda1 (0.3 μM) and histamine (His; 100 μM) in SW982 cells treated with either si-Piezo1 or sc-RNA; (**C**,**D**). A summary of the peak change (ΔCa^2+^) in cells treated with either si-Piezo1 or sc-RNA; (**E**) The effect of *PIEZO1* knockdown on SW982 cell-viability; (**F**) SW982 cell-viability after treatment with Yoda1 at a concentration range between 0.01 and 3 μM for 48 h. Pooled data are averaged and expressed as mean ± SEM. Statistical significance was established using Student’s *t*-test. * *p* < 0.05 and ** *p* < 0.01 compared with each corresponding control group. ‘ns’ indicates no significance. The numbers in parentheses indicate the number of independent experiments.

To test the function of PIEZO1 in synovial sarcoma, we next knocked down *PIEZO1* in SW982 cells using stealth small interfering RNA (siRNA). As shown in Figure 4A, the level of *PIEZO1* mRNA in SW982 cells treated with *PIEZO1-*specific siRNA (si-Piezo1) for 72 h was significantly reduced compared with sc-RNA (see also Supplementary Figure S2). SW982 cells treated with si-Piezo1 were then exposed to Yoda1 and histamine (His) while monitoring their Ca^2+^ responses (Figure 4B–D). Compared with SW982 cells with sc-RNA, the Ca^2+^ response of SW982 cells treated with si-Piezo1 to 0.3 μM Yoda1 was significantly reduced (Figure 4C), while the response to 100 μM His was unchanged (Figure 4D). This suggests that PIEZO1 is not functional in SW982 cells treated with si-Piezo1. To use these cells, we compared the cell-viability of SW982 cells with and without functional PIEZO1. As shown in Figure 4E, *PIEZO1* knockdown in SW982 cells effectively reduced cell-viability, suggesting that this protein is a functional regulator of cell-viability in SW982 synovial sarcoma cells. In contrast, Yoda1-induced activation of PIEZO1 had little effect on cell-viability.

## 3. Discussion

In this study, we found that synovial sarcoma SW982 cells express PIEZO1, which is effectively activated by Yoda1 in a concentration-dependent manner. Knocking down *PIEZO1* expression reduced SW982 cell-viability as well as Yoda1-induced Ca^2+^ response, suggesting that PIEZO1 is a potential regulator of cancer cell-viability.

PIEZO1 is a mechano-sensor that is effectively activated by a chemical agonist Yoda1 [6]. Yoda1 was shown to substantially activate mouse Piezo1 in red blood cells [22] and vascular endothelial cells [20]. In the present study, we showed that Yoda1 evoked Ca^2+^ responses in HEK-Piezo1 and SW982 cells, both of which were abolished in the absence of extracellular Ca^2+^. In addition, Yoda1 caused the activation of cation channel currents in both cell types, strongly suggesting that synovial sarcoma SW982 cells express functional PIEZO1. Interestingly, we found the apparent EC_50_ of Yoda1 against SW982 cells to be less than 1 μM (Figure 1E), while the apparent EC_50_ for human PIEZO1 expressed in HEK cells was reported to be 26.6 μM [6]. The reason for this discrepancy is not clear, but the different experimental conditions may affect the biological response to Yoda1. Consistent with our results, Yoda1 at less than 1 μM effectively activated Piezo1 in mouse vascular endothelial cells [20]. Because Yoda1 is highly lipophilic [6], it is possible that this physical property affects experimental assays. Nevertheless, Yoda1 is a useful chemical activator for PIEZO1. The conductance of Yoda1-induced intrinsic channel was identical to that of mechanical stress sensitive channel (21.6 pS, Figure 2C): Yoda1 at 3 μM and 10 μM had little effect on HEK-hTRPC4 cells and HEK-cont cells (Supplementary Figure S1).

It has been shown that mechanical stress affects cancer cell migration and invasion [23]. Moreover, the blockade of mechano-sensitive channels by tarantula toxin GsMTx-4 inhibited the motility of human breast cancer cell line MCF-7 [24]. Because *PIEZO1* is highly expressed in MCF-7 cells and is substantially inhibited by GsMTx-4, it is proposed that PIEZO1 is a potential regulator of cancer cell motility. In contrast, the loss of *PIEZO1* expression caused increased cell migration and metastasis in lung cancer cells [25]. In the present study, the knockdown of *PIEZO1* significantly reduced the cell-viability of SW982 cells. However, it is not clear if channel activation itself plays a key role in the regulation of cell-viability. In fact, Yoda1-induced activation of PIEZO1 had little effect on SW982 cell-viability, suggesting that PIEZO1 channel function is not involved in this process. The deletion of *PIEZO1* reduces cell adhesion [19] and thus may affect cell-viability.

Our recent study has demonstrated that the activation of heteromeric TRPC4 and TRPC1 (TRPC4/C1) causes a potent cytotoxic effect on SW982 cells mediated via Na^+^ loading [21], suggesting that heteromeric TRPC4/C1 is a promising target of anti-tumor agents against synovial sarcoma. Because cell-viability was effectively inhibited in SW982 cells lacking functional PIEZO1 in the present study, PIEZO1 is a potential novel target against synovial sarcoma. In addition, it has been shown that PIEZO1 activation by cell stretch induces the apoptosis of human chondrocytes in osteoarthritis [26]. *PIEZO1* is ubiquitously expressed in neuronal and non-neuronal organs. A global loss of Piezo1 in mouse is lethal during mid-gestation because of disrupted development of vasculature [1,2]. A smooth muscle cell-specific loss of *PIEZO1* causes the deficit of arterial remodeling upon hypertension [27]. Moreover, endothelial PIEZO1 regulates blood pressure in mice both with [20] and without exercise [28]. Therefore, the functional significance of application anti-PIEZO1 agents is rather complicated [3] and further extensive studies are required.

## 4. Materials and Methods

### 4.1. Reagents

The following reagents were used: 2-[5-[[(2,6-Dichlorophenyl)methyl]thio]-1,3,4-thiadiazol-2-yl]-pyrazine (Yoda1, Tocris Bioscience, Bristol, UK), (-)englerin-A (EA, AppliChem, Darmstadt, Germany), acetylcholine (ACh, Wako, Osaka, Japan), and histamine (His, Wako). Each reagent was dissolved in the vehicle recommended by the manufacturer.

### 4.2. Cell Culture

Human synovial sarcoma SW982 (SW982, American Type Culture Collection, Manassas, VA, USA) and human embryonic kidney 293 cell lines (HEK, Health Science Research Resources Bank, Osaka, Japan) were maintained in culture media recommended by the manufacturers [21]. All culture media were supplemented with 10% heat-inactivated FCS (GIBCO, Waltham, MA, USA), streptomycin (100 μg/mL, Meiji Seika Pharma Co., Ltd., Tokyo, Japan), and penicillin G (100 U/mL, Meiji Seika Pharma Co., Ltd.).

### 4.3. Recombinant Expression of PIEZO1 in HEK Cells

Partially confluent HEK cells (40–60% confluency) were transfected with the pcDNA3.1 (for Ca^2+^ measurements) and pIRES2-AcGFP1 (for patch-clamp experiments) plasmids containing human *PIEZO1* and *TRPC4*, respectively, using Lipofectamine 3000 (ThermoFisher Scientific, Yokohama, Japan). All constructs were verified by sequencing. Cells were used in experiments within 48 h after transfection.

### 4.4. Quantitative PCR and RT-PCR

Real-time quantitative PCR was performed with SYBR Green chemistry on a Thermal Cycler Dice Real Time System (Takara Bio, Inc., Kusatsu, Japan) as described previously [21]. Transcriptional quantification of gene products was performed relative to *β*-*ACTIN*. Each cDNA sample was tested in triplicate. The program used for quantitative PCR amplification consisted of a 30 s activation of Ex Taq™ DNA polymerase at 95 °C, a 15 s denaturation step at 95 °C, a 60 s annealing and extension step at 60 °C (for 45 cycles), and a dissociation step (15 s at 95 °C, 30 s at 60 °C, and 15 s at 95 °C). The oligonucleotide sequences of primers specific for human *PIEZO1* and β*-ACTIN* are: TAGCCATTACTACCTGCACGTC (forward), TGCGGTGAAAGTCAATGCTC (reverse) and ACCGAGCGCGGCTACA (forward), CAGCCGTGGCCATCTCTT (reverse), respectively. RT-PCR amplification for *PIEZO1* and *β-ACTIN* was performed as followings. The thermal cycler program used for PCR amplification included a 30 s denaturation step at 94 °C, a 30 s annealing step at 55 °C, and a 30 s primer extension step at 72 °C for 29 and 22 cycles for *PIEZO11* and *β-ACTIN*, respectively, using an ABI 2720 thermal cycler (Applied Biosystems, Foster City, CA, USA). The amplified products were separated on 1.5% agarose gels in Tris acetate/EDTA buffer, visualized with 1 μg/mL ethidium bromide, and assessed on FAS III (TOYOBO, Osaka, Japan).

### 4.5. Patch-Clamp Experiments

Whole-cell current and single-channel recording experiments were performed as described previously [29,30]. The resistance of pipettes was 3–5 MΩ when filled with pipette solution. A Cs^+^ rich pipette solution containing 110 mM Cs-aspartate, 30 mM CsCl, 1 mM MgCl_2_, 10 mM HEPES, 1 mM EGTA, and 2 mM Na_2_ATP was adjusted to pH 7.2 with CsOH. Membrane currents and voltage signals were amplified with an EPC-800 amplifier (HEKA, Lambrechit, Germany) and digitized at 10 KHz using an analogue-digital converter (PCI6229, National Instruments Japan, Tokyo, Japan) driven by WinWCPV4.5 and WINEDR3.38 for data acquisition and analysis for whole-cell currents and cell-attached single channel currents, respectively (developed by John Dempster, University of Strathclyde, Glasgow, UK). The liquid junction potential between the pipette and bath solutions (−10 mV) was corrected when the aspartate rich pipette solution was used. A ramp voltage protocol from −110 mV to +90 mV for 400 ms was applied every 5 s from a holding potential of −10 mV. A leak current component was not subtracted from the recorded currents. A standard HEPES-buffered bathing solution (SBS: 137 mM NaCl, 5.9 mM KCl, 2.2 mM CaCl_2_, 1.2 mM MgCl_2_, 14 mM glucose, 10 mM HEPES, adjusted to pH 7.4 with NaOH) was used. In the cell-attached patch experiments, the pipette contained SBS supplemented with 10 mM TEA. To set a resting membrane potential around 0 mV in the cell-attached patch configuration, high K^+^ bathing solution was used (140 mM KCl, 2.2 mM CaCl_2_, 1.2 mM MgCl_2_, 14 mM glucose, 10 mM HEPES, adjusted to pH 7.4 with NaOH). All experiments were performed at 25 ± 1 °C.

### 4.6. Measurement of Ca^2+^ Fluorescence Ratio

Cells were loaded with 10 μM Fura2-AM (Dojindo, Kumamoto, Japan) in SBS for 30 min at 24–26 °C, superfused with SBS for 10 min, and Fura-2 fluorescence signals were measured at 0.1 Hz using the Argus/HisCa imaging system (Hamamatsu Photonics, Hamamatsu, Japan) driven by Imagework Bench 6.0 (INDEC Medical Systems, Santa Clara, CA, USA). In each analysis, the whole cell area was chosen as the region of interest to average the fluorescence ratio.

### 4.7. Specific Knockdown of PIEZO1 by RNA Interference

The sequence of the stealth short interfering RNA (si-RNA) duplex oligonucleotides against human *PIEZO1* (si-Piezo1, Dharmacon, part of GE Healthcare, Lafayette, CO, USA) is 5′-GCCUCGUGGUCUACAAGAUTT-3′ for the sense strand and 5′-AUCUUGUAGACCACGAGGCTT-3′ for the antisense strand. As a negative control for the si-RNA treatment, Non-Targeting siRNA #1 (sc-RNA, Dharmacon) was used. The cells grown in a 35-mm dish or a 24-well plate were washed with fresh culture medium without antibiotics 3 h prior to transfection. The si-Piezo1 or sc-RNA (20 µM, 2 µL for the 35-mm dish and 0.5 μL for the 24-well plate, respectively) and Lipofectamine RNAiMAX (2.5 µL for the 35-mm dish and 0.63 μL for the 24-well plate, respectively; Invitrogen) were diluted in 250 µL (35-mm dish) and 62.5 μL (24-well plates) Opti-MEM (Invitrogen), respectively, mixed together and incubated for 20 min at room temperature for complex formation. The entire mixture was added to the cells, resulting in a final concentration of 20 nM for both si-Piezo1 and sc-RNA. The cells were incubated for 72–96 h in a CO_2_ chamber.

### 4.8. WST-1 Cell-Viability Assay

Cells were seeded onto 24-well plate 24 h prior to WST-1 measurements (1 × 10^4^ SW982 cells were used for analysis). The cell-viability reagent WST-1 (Roche Applied Science, Penzberg, Germany) was used in accordance to the manufacturer’s instructions [21]. Reduction of the tetrazolium salt WST-1 to formazan by mitochondrial dehydrogenases was determined by measuring absorbance at 450 nm (DXT880 Multimode Detector, Beckman Coulter, Brea, CA, USA). Cell death resulting in the loss of mitochondrial dehydrogenase activity was inferred from a decrease in the yield of this reaction. Background absorbance at the reference wavelength 620 nm was subtracted. Each sample was tested in duplicate or triplicate and pooled data were summarized across independent experiments.

### 4.9. Statistical Analyses

Data are expressed as the mean ± SEM. Statistical significance between two groups and across multiple groups was examined using unpaired Student’s *t*-test and ANOVA test, respectively, both two-tailed (Origin J9.1, LightStone, Tokyo, Japan). For all tests, *p* values below 0.05 were considered statistically significant.

## 5. Conclusions

Our results provide new insights into PIEZO1 function showing that this mechno-sensing channel is highly expressed in synovial sarcoma SW982 cells and can regulate their cell-viability. The molecular mechanisms underlying the anti cell-viability in synovial sarcoma without PIEZO1 are yet to be elucidated and potentially involve the inhibition of cell adhesion.

## Supplementary Materials

The following are available online at http://www.mdpi.com/1422-0067/19/5/1452/s1, Figure S1: Yoda1 has no effect on membrane currents in HEK-hTRPC4 and HEK-cont cells. (A) HEK-hTRPC4 cells were voltage-clamped in whole-cell configuration mode and treated with 3 μM Yoda1. After the wash-out of Yoda1, the cells were exposed to 100 nM EA. Left panel: Ramp waveform pulses from −110 to +90 mV for 400 ms were applied every 5 s and the peak amplitude of membrane currents at −80 and +80 mV was plotted against time. Arrows denote the time at which each I-V was detected. Right panel: A typical I-V exhibited before and after the application of 3 μM Yoda1, and before and after the application of 100 nM EA. (B) A summary of the peak amplitudes of membrane currents evoked at −80 and +80 mV in the presence of 3 μM Yoda1 and 100 nM EA in HEK-hTRPC4 cells, and 10 μM Yoda1 in HEK-cont cells. Pooled data were averaged and expressed as mean ± SEM. The numbers in parentheses indicate the number of independent experiments. Figure S2: The mRNA expression of *PIEZO1* and *β-ACTIN* was determined with RT-PCR in SW982 cells treated with sc-RNA and si-Piezo1. Three independent samples were tested.
