# Supplementary material for: PIEZO1 Channel Is a Potential Regulator of Synovial Sarcoma Cell-Viability
Source: Int J Mol Sci. 2018 May 14;19(5):1452. doi: 10.3390/ijms19051452 (PMC5983681; doi:10.3390/ijms19051452)
Supplement: Supplementary file 1 [file ijms-19-01452-s001.pdf]

International Journal of
Molecular Sciences

MDPI

Supplementary Materials

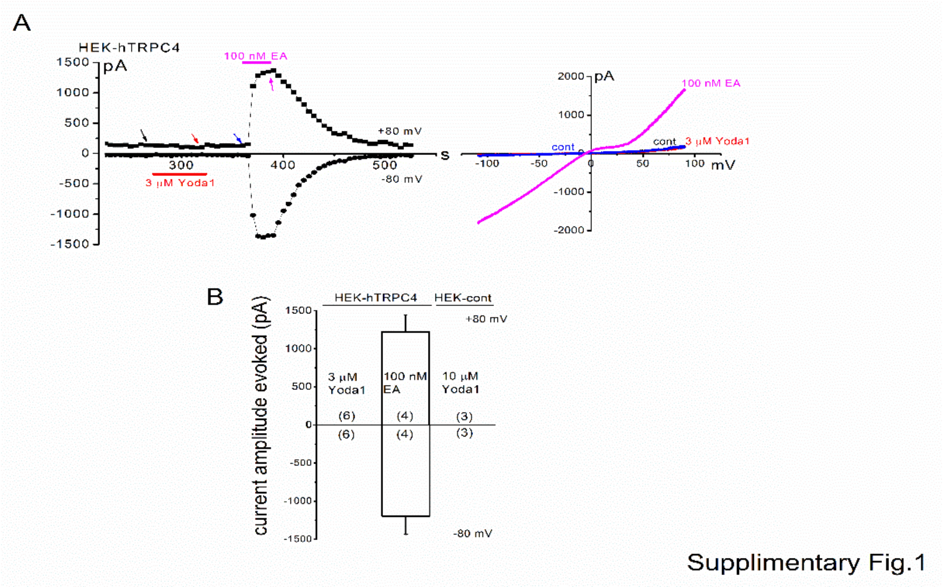

Figure S1: Yoda1 has no effect on membrane currents in HEK-hTRPC4 and HEK-cont cells. (A) HEK-hTRPC4 cells were voltage-clamped in whole-cell configuration mode and treated with 3 μM Yoda1. After the wash-out of Yoda1, the cells were exposed to 100 nM EA. Left panel: Ramp waveform pulses from −110 to +90 mV for 400 ms were applied every 5 s and the peak amplitude of membrane currents at −80 and +80 mV was plotted against time. Arrows denote the time at which each I-V was detected. Right panel: A typical I-V exhibited before and after the application of 3 μM Yoda1, and before and after the application of 100 nM EA. (B) A summary of the peak amplitudes of membrane currents evoked at −80 and +80 mV in the presence of 3 μM Yoda1 and 100 nM EA in HEK-hTRPC4 cells, and 10 μM Yoda1 in HEK-cont cells. Pooled data were averaged and expressed as mean ± SEM. The numbers in parentheses indicate the number of independent experiments.

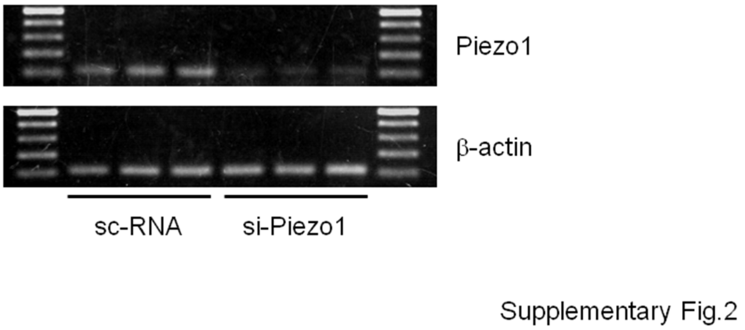

Figure S2. The mRNA expression of *PIEZO1* and *β-ACTIN* was determined with RT-PCR in SW982 cells treated with sc-RNA and si-Piezo1. Three independent samples were tested.
